# Critical role of the finger loop in arrestin binding to the receptors

**DOI:** 10.1371/journal.pone.0213792

**Published:** 2019-03-15

**Authors:** Chen Zheng, Jonas Tholen, Vsevolod V. Gurevich

**Affiliations:** 1 Department of Pharmacology, Vanderbilt University, Nashville, United States of America; 2 University of Applied Sciences Emden/Leer, Emden, Germany; Indian Institute of Technology Kanpur, INDIA

## Abstract

We tested the interactions with four different G protein-coupled receptors (GPCRs) of arrestin-3 mutants with substitutions in the four loops, three of which contact the receptor in the structure of the arrestin-1-rhodopsin complex. Point mutations in the loop at the distal tip of the N-domain (Glu157Ala), in the C-loop (Phe255Ala), back loop (Lys313Ala), and one of the mutations in the finger loop (Gly65Pro) had mild variable effects on receptor binding. In contrast, the deletion of Gly65 at the beginning of the finger loop reduced the binding to all GPCRs tested, with the binding to dopamine D2 receptor being affected most dramatically. Thus, the presence of a glycine at the beginning of the finger loop appears to be critical for the arrestin-receptor interaction.

## Introduction

G-protein coupled receptors (GPCRs) are the largest family of signaling proteins in mammals, with ~500 different subtypes in dolphins, ~800 in primates, and more than 3,400 in elephants (sevens.cbrc.jp). GPCRs are involved in almost every aspect of life activity by mediating most cellular responses to hormones, neurotransmitters, odorants, light, etc. [1].

All GPCRs share a common seven transmembrane (7TM) α-helical segments linked by extracellular loops (ECLs) and intracellular loops (ICLs). ECLs, ICLs, as well as the extracellular N-termini and intracellular C-termini are diverse in sequence and length [2,3]. The activation of GPCRs induced by external stimuli leads to the activation of many molecules of heterotrimeric G-proteins to amplify signal. Ultimately GPCR signaling via G proteins is terminated by receptor phosphorylation by specific GPCR kinases (GRKs; of which most mammals only have seven, with some nocturnal rodents having only six [4]), and specific binding of arrestins to active phosphorylated receptors [5,6]. Mammals express even fewer arrestin subtypes than GRKs, the total of four, two out of which are specialized visual that are expressed in retinal photoreceptors and quench photopigment signaling [7,8]. In contrast, non-visual subtypes arrestin-2 and -3 (also known as β-arrestin1 and 2; note that we use systematic names of arrestin proteins, where the number after the dash indicates the order of cloning: arrestin-1 (historic names S-antigen, 48 kDa protein, visual or rod arrestin), arrestin-2 (β-arrestin or β-arrestin1), arrestin-3 (β-arrestin2 or hTHY-ARRX), and arrestin-4 (cone or X-arrestin)), are ubiquitously expressed and bind hundreds of non-visual GPCRs.

Identified >800 GPCRs in humans account for ~ 3% of coding genes [9]. Abnormal GPCR functions due to mutations are associated with a wide variety of congenital disorders [10–12]. Currently, about 30% of clinically used drugs target various GPCRs [2]. For gain-of-function GPCR mutations, engineered enhanced arrestins with higher ability to bind phosphorylated and unphosphorylated receptors may have therapeutic potential, as these proteins can reduce the excessive signaling of overactive GPCRs [12,13].

Considering that visual and non-visual arrestins are highly conserved with only a few residues different [8], the high specificity of visual arrestin for rhodopsin might be tuned by those key residues. Previous study suggests that receptor preference of visual arrestin-1 and non-visual arrestin-2 can be switched by the exchange of ten residues on the receptor-binding interface [14]. A double mutation in arrestin-3 yielded up to 50-fold preference for particular receptors over others [15], indicating the possibility of narrowing arrestin-3 receptor preference and constructing receptor-specific variants. Recent structural studies of the arrestin-1 complex with rhodopsin revealed that several arrestin elements directly participate in receptor binding [16,17]. Here we test how mutations in these regions of arrestin-3 affect its binding to the four model GPCRs: β2-adrenergic, M2 muscarinic, and D1 and D2 dopamine receptors.

## Materials and methods

### Materials

Restriction endonucleases and other DNA modifying enzymes were from New England Biolabs (Ipswich, MA). Cell culture reagents and media were from GIBCO (Gaithersburg, MD). Transfection reagent TransHi was from FormuMax (Sunnyvale, CA). Coelenterazine h was from NanoLight (Pinetop, AZ). All plasmid DNAs were prepared by Qiagen (Germantown, MD) Plasmid Maxi kit. All other reagents and chemicals were purchased from Amresco (Solon, OH) or Sigma-Aldrich (St Louis, MO).

### Plasmid constructs

All arrestin mutants were N-terminally tagged with Venus, whereas the receptors were C-terminally tagged with *Renilla* luciferase variant 8 (*R*Luc8), as described [14,15].

### Bioluminescence resonance energy transfer (BRET) assay

BRET-based assays [18–21] with Venus as the acceptor and *R*Luc8 as the donor were used to measure the binding of Venus-tagged arrestins to the M2 muscarinic acetylcholine receptor (M2R-*R*Luc8), β2-adrenergic receptor (β_2_AR-*R*Luc8), dopamine D1 (D1R-*R*Luc8) and D2 (D2R-*R*Luc8) receptors. The long isoform of D2R was used in this study, as in previous ones [15,22]. HEK293 arrestin-2/3 KO cells [23,24] (a kind gift of Dr. A. Inoue, Tohoku University, Japan) were transfected using TransHi according to the manufacturer’s instructions (3 μl of TransHi /1 μg of DNA) in 6-well plates.

We have previously determined the amounts of plasmid DNA that produce sufficient excess of arrestin-3 over receptor that saturates the BRET signal [15,25,26]. 48 h post-transfection, cells expressing similar levels of receptor and arrestins (S1 Fig) were transferred into 96-well plate, then appropriate agonists (10 μM) were added: carbachol (carbamoylcholine) for M2R, isoproterenol for β_2_AR, dopamine for D1R, and quinpirole for D2R. Coelenterazine h was added immediately after agonist as luciferase substrate at 5 μM. The net BRET (the difference in BRET in the presence and absence of an agonist) was measured as previously described [15,26,27].

### Co-immunoprecipitation

HEK293 arrestin 2/3 KO cells were seeded into 6-well plate 24 h prior to transfection. Cells were transfected with indicated Ve-Arr3 mutants and HA-D2R using TransHi. The cells were serum-starved overnight one day prior to test. At 48 h post-transfection, cells were washed with PBS and treated with 10 μM quinpirole for 10 min. Then cells were immediately lysed on ice in 400 μL of lysis buffer containing 50 mM Tris pH 7.5, 150 mM NaCl, 1% NP-40, 1 mM Benzamidine, 1 mM PMSF. The lysates were centrifugated for 10 min at 11, 000xg to pellet cell debris, and the supernatants were used for immunoprecipitation. A total of 400 μg protein from each transfection were pre-cleared with 25 μL protein G agarose (Millipore, MO) in binding buffer (25 mM HEPES, pH 7.3, 150 mM NaCl, 1 mM TCEP), followed by incubation for 2 h with 2 μg Anti-HA antibody (Roche, NJ). The immunoprecipitates were incubated with 25 μL of protein G agarose beads overnight at 4°C. The next day the beads were sedimented by centrifugation for min at 4°C at 5, 000 xg and washed three times with 500 μL binding buffer. Bound proteins were eluted with 50 μL of SDS sample buffer. Cell lysates and immunoprecipitated proteins were subjected to Western blot using anti-HA and anti-GFP antibodies, peroxidase-coupled secondary antibodies (Jackson Immuno, PA), and SuperSignal WestPico reagent (ThermoFisher, IL). Developed blots were visualized by LI-COR C-DiGit Blot Scanner (LI-COR, NE). Bands were quantified using QuantityOne software (Bio-Rad, CA). Non-specific binding to beads (in the absence of HA-D2R bait) was subtracted.

### Data analysis and statistics

BRET data were analyzed, as described [15,22]. Statistical significance (*p* < 0.05) was determined using one-way analysis of variance (ANOVA) with Dunnett's multiple comparison test using GraphPad Prism software. P values<0.05 were considered statistically significant and indicated, as follows: *p<0.05; ***p<0.001; ****p<0.0001.

## Results

### Selection of receptor-specific mutants

Crystal structures indicate high similarity of the basal state of all vertebrate arrestins [28–33]. Receptor-binding surface of all arrestins was localized to the concave sides of the two domains and the central crest on the receptor-binding side of the molecule by different labs using a variety of methods [34–40]. The crystal structure of the arrestin-1 complex with activated phosphorylated rhodopsin (P-Rh*) has identified two interaction interfaces in arrestins largely responsible for the binding of non-phosphorylated parts of the receptor [16,17]. The first interface consists of the finger loop (residues G65-S75 in arrestin-3, corresponding to G68-S78 in arrestin-1), which was shown to be the key region for receptor binding of arrestin-3 [41]. Second interface consists of the middle loop (Q131-A140 in arrestin-3; Q133-S142 in arrestin-1), C-loop (residues C243-Q247 in arrestin-3; V247-Y254 in arrestin-1, residue Y251 region at the central loop in the arrestin C-domain) and back loop (K313 loop, K310 in arrestin-1, R319/R322 in mouse/human arrestin-1). The P-Rh*-arrestin-1 complex structure shows that the middle and C-loop are close in the basal state but move away from each other upon activation, opening a cleft in the central crest. The shift of the back loop apparently twists the arrestin C-domain, which allows back loop (K319 and T320) interact with TM5/6 of rhodopsin through hydrogen bonds, and allows the 157-loop (residue D162 in arrestin-1, E157 in arrestin-3) move closer to the finger loop [16,36,42].

**Fig 1:**
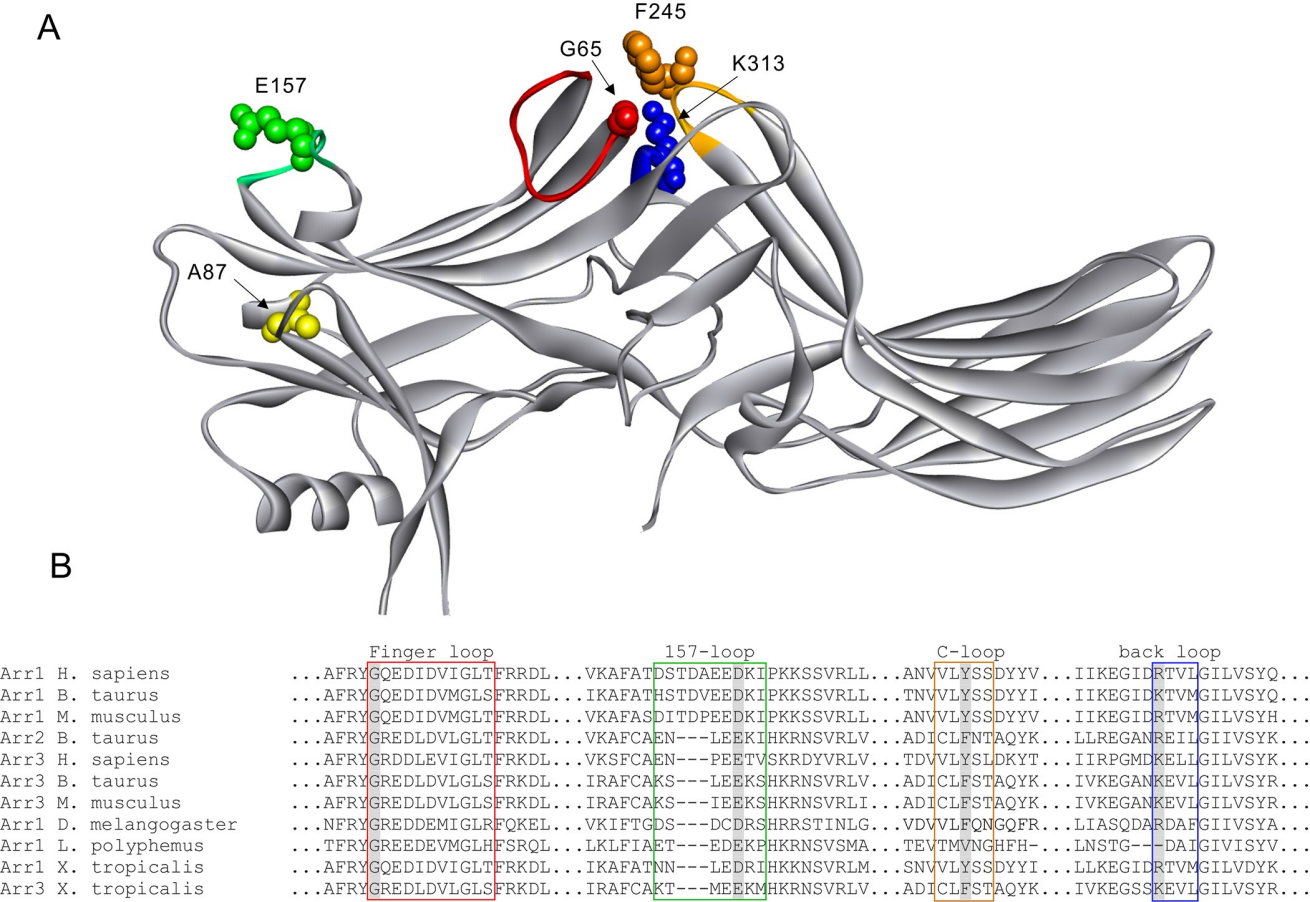
Arrestin residues mutated in this study. **A**. Crystal structure of arrestin-3 (Protein Data Bank entry 3P2D [33]) with selected mutations indicated. Arrestin elements are colored, as follows: (Red: finger loop; Green: 157-loop; Yellow: C-loop; Blue: back-loop). **B**. Sequence alignment of elements containing selected mutations in arrestin-3 and other subtypes from different species. Shaded residues in each loop are the mutations selected in this study.

Sequence comparison identifies a few residue differences in those loops at the interfaces. Most of these residues are exposed in the basal state, so that they might be directly engaged by the receptor (Fig 1A). Based on these data, we substituted several residues in arrestin-3 targeting those loops that were expected to be flexible. For example, G65 (in bovine arrestin-3) is the first residue of the finger loop and it is conserved in all arrestin subtypes (Fig 1B). As finger loop is highly flexible, the small size of glycine might allow finger loop movement during activation process [43]. We made the G65P mutant because proline provides more rigidity than glycine, while also breaking any secondary structure. We also tested the G65 deletion (ΔG65) because of high conservation of this residue in arrestin evolution (Fig 1B) [7,8].

E157, located on the flexible 157-loop, is conserved in non-visual arrestins, but replaced with Asn in the corresponding position of arrestin-1 where it interacts with the TM6 of the rhodopsin via a hydrogen bond (Fig 1B) [44]. We chose to introduce E157A mutation to break the potential hydrogen bond formation by this side chain.

In arrestin-1 both Y251 (F245 in arestin-3) in the C-loop and K319 (K313 in arrestin-3) in the back loop participate in rhodopsin binding via interaction with ICL2 and TM5, respectively, suggesting their importance in stabilizing the arrestin-receptor complex. Phe is conserved in non-visual arrestins at the position. However, the substitution of Phe with Tyr at this site in arrestin-2 does not change its receptor preference [14]. So, we chose the F245A mutation to eliminate this aromatic side chain. K313A mutation was chosen to eliminate positively charged side chain.

### Highly conserved G65 in the finger loop is essential for receptor binding

To measure arrestin-3 interaction with GPCRs, we used bioluminescence resonance energy transfer (BRET) in HEK293 arrestin2/3 KO cells [22] co-transfected with Venus-tagged arrestin-3 (Ve-Arr3) and *Renilla* luciferase-tagged (*R*Luc) receptors M2R, β_2_AR, D1R and D2R. In all tests, we used arrestin-3 KNC mutant as negative control. The KNC mutant does not bind GPCRs because 12 key receptor-binding residues in it were replaced with Ala [25,26]. All mutations were introduced on arrestin-3 A87V background. The A87V mutation makes arrestin-3 N-domain more rigid [15]. This substitution likely enhances receptor specificity of arrestins [30] without significantly affecting the binding to any GPCR used in this study [15].

**Fig 2:**
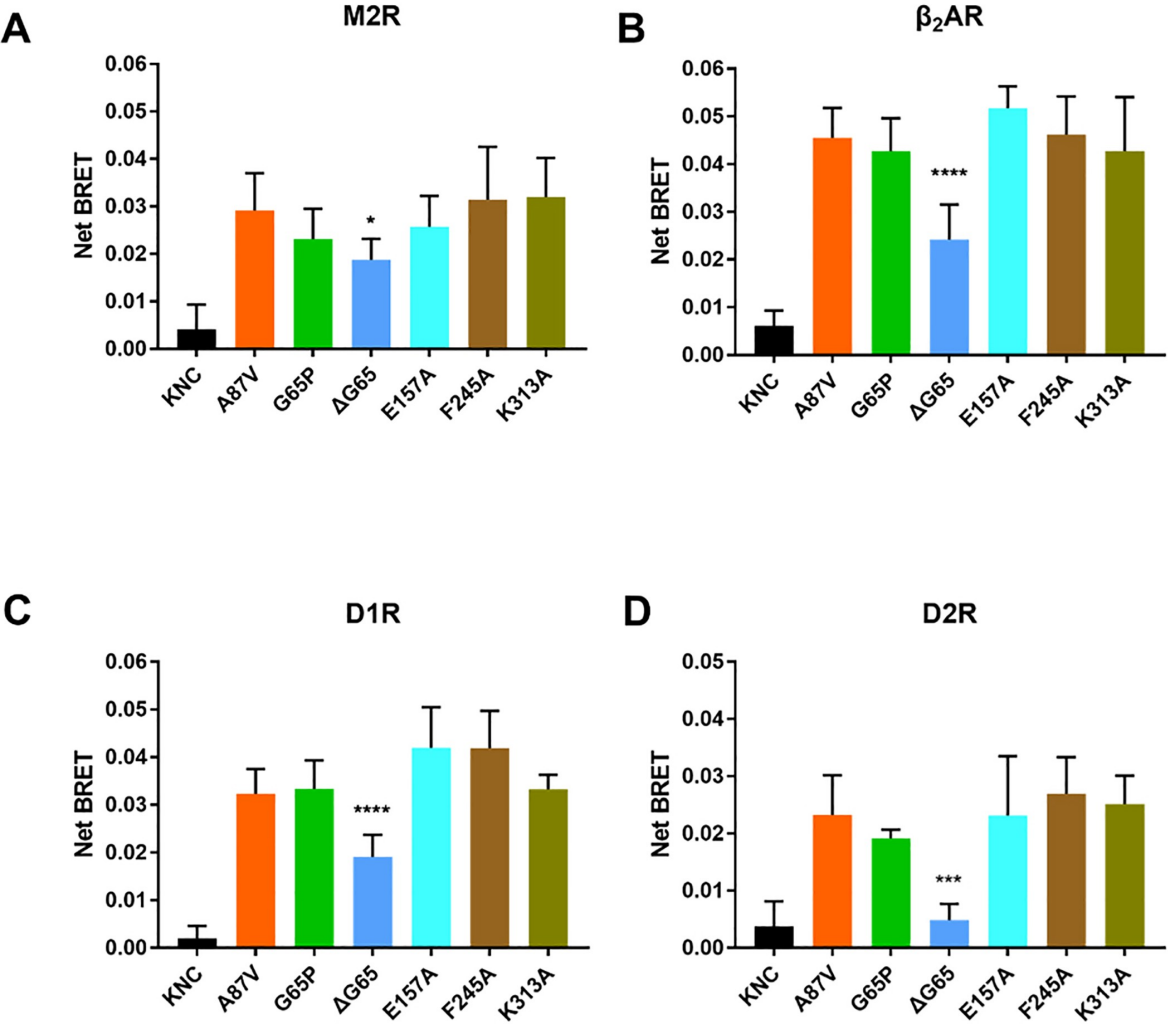
Substitution of selected residues in arrestin-3 loops differentially affects agonist-induced binding to individual GPCRs. BRET between Venus-tagged arrestin-3 and luciferase-tagged human M2R (**A**), β_2_AR(**B**), D1R(**C**), D2R(**D**) in HEK293 arrestin 2/3 KO cells. Net BRET was calculated by subtracting basal BRET (no agonist) from agonist-induced BRET. Average BRET at 10 and 15 min (means ± S.E.M.) from at least three independent experiments is shown for each arrestin-receptor pair. Each experiment was performed in quadruplicate. Statistical significance was determined using one-way ANOVA, followed by Dunnett's post-hoc test with correction for multiple comparisons. *, p< 0.05; ***, p< 0.001; ****, p<0.0001, as compared to A87V base mutant.

Base mutant A87V showed a robust binding upon agonist stimulation, while KNC mutant consistently showed extremely low binding, as expected (Fig 2). Four mutations did not significantly affect arrestin-3 binding to any of the four GPCRs tested: G65P, E157A, F245A and K313A (Fig 2). However, ΔG65 dramatically reduced the binding to all four receptors (Fig 2). The magnitude of the reduction varied from ~30% to ~80%. In particular, ΔG65 dramatically reduced arrestin-3 binding to D2R, almost to the level observed with the KNC mutant. Considering that receptor specificity of arrestins appears to be determined by several residues [15,27], it is surprising that a single mutation can make such a dramatic difference. More importantly, although ΔG65 reduced binding to all of four GPCRs, this mutation affected D2R binding to a much greater extent than others, suggesting that stability of D2R-arrestin complex is more dependent on the receptor interaction with the finger loop of arrestin.

### Receptor binding might be sterically hindered by the deletion of G65

**Fig 3:**
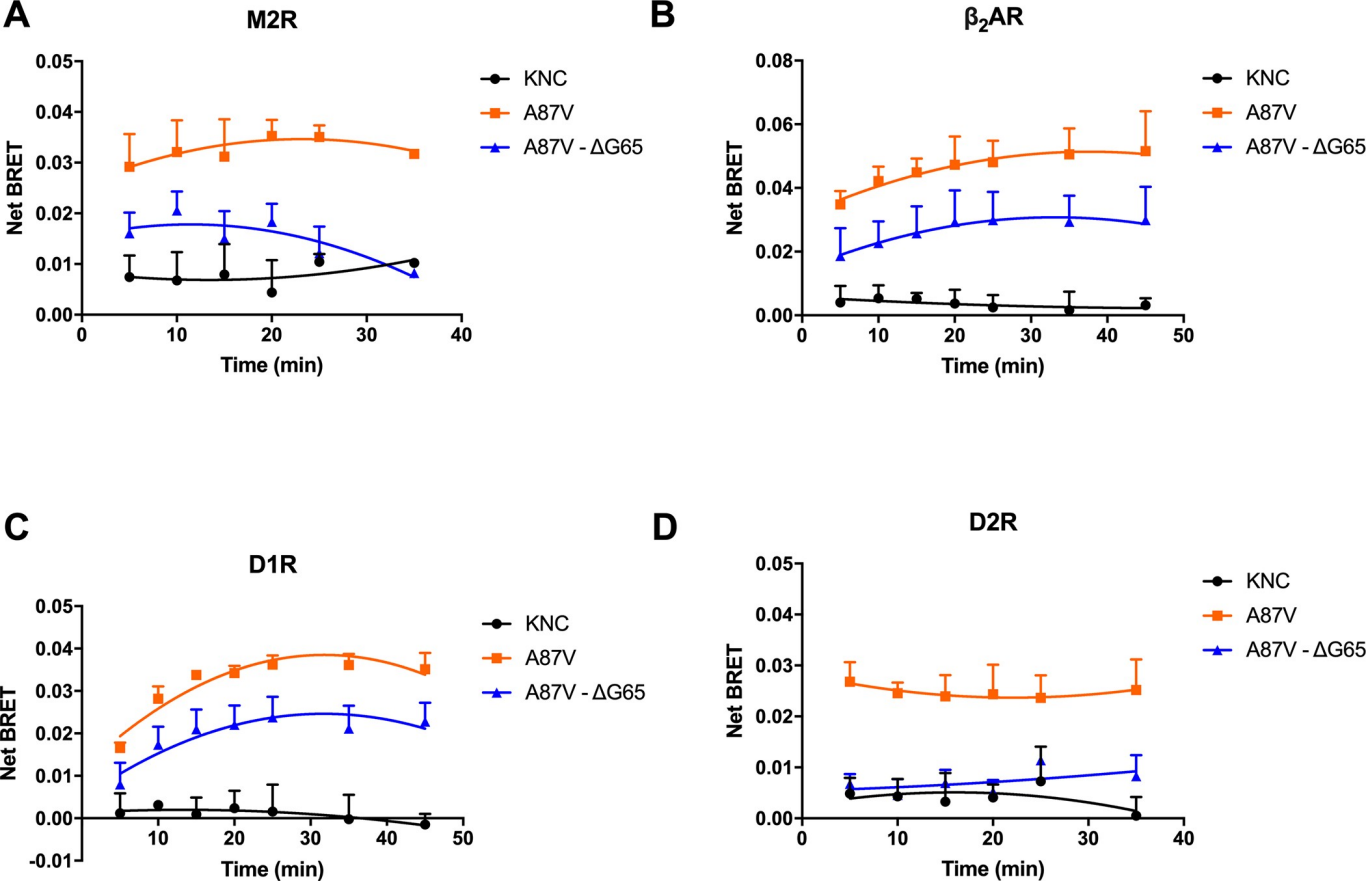
Time course of the interaction of selected arrestins with indicated receptors. Net BRET of selected Venus-tagged arrestin-3 mutants measured at indicated time points with luciferase-tagged M2R (**A**), β_2_AR(**B**), D1R(**C**), D2R(**D**) is shown (means ± S.E.M.). Note similar time dependence of A87V and ΔG65 mutant binding to the receptors, even though ΔG65 consistently demonstrates much lower binding.

To elucidate the mechanism of action of ΔG65 mutation, we tested the time dependence of net BRET, comparing ΔG65 to non-binding KNC and robustly binding A87V base mutant. For all four receptors, A87V showed increased net BRET in the first 10–15 min after the addition of the agonist, which subsequently decreased at 25–30 min. In contrast, KNC mutant consistently showed low binding at all time points (Fig 3A–3D). The time course of the binding of mutant with the deletion of G65 was similar to that of A87V, while being consistently lower than the binding of A87V at all time points. Since ΔG65 mutant never reaches the BRET value demonstrated by A87V, this mutation likely affects receptor binding by reducing the stability of the arrestin-receptor complex, i.e., by increasing the rate of arrestin dissociation, so that there is less arrestin bound at any given moment, without significant changes in the kinetics of the initial arrestin-3 recruitment to receptors (Fig 3).

### Independent assessment of arrestin-receptor interaction by co-immunoprecipitation

**Fig 4:**
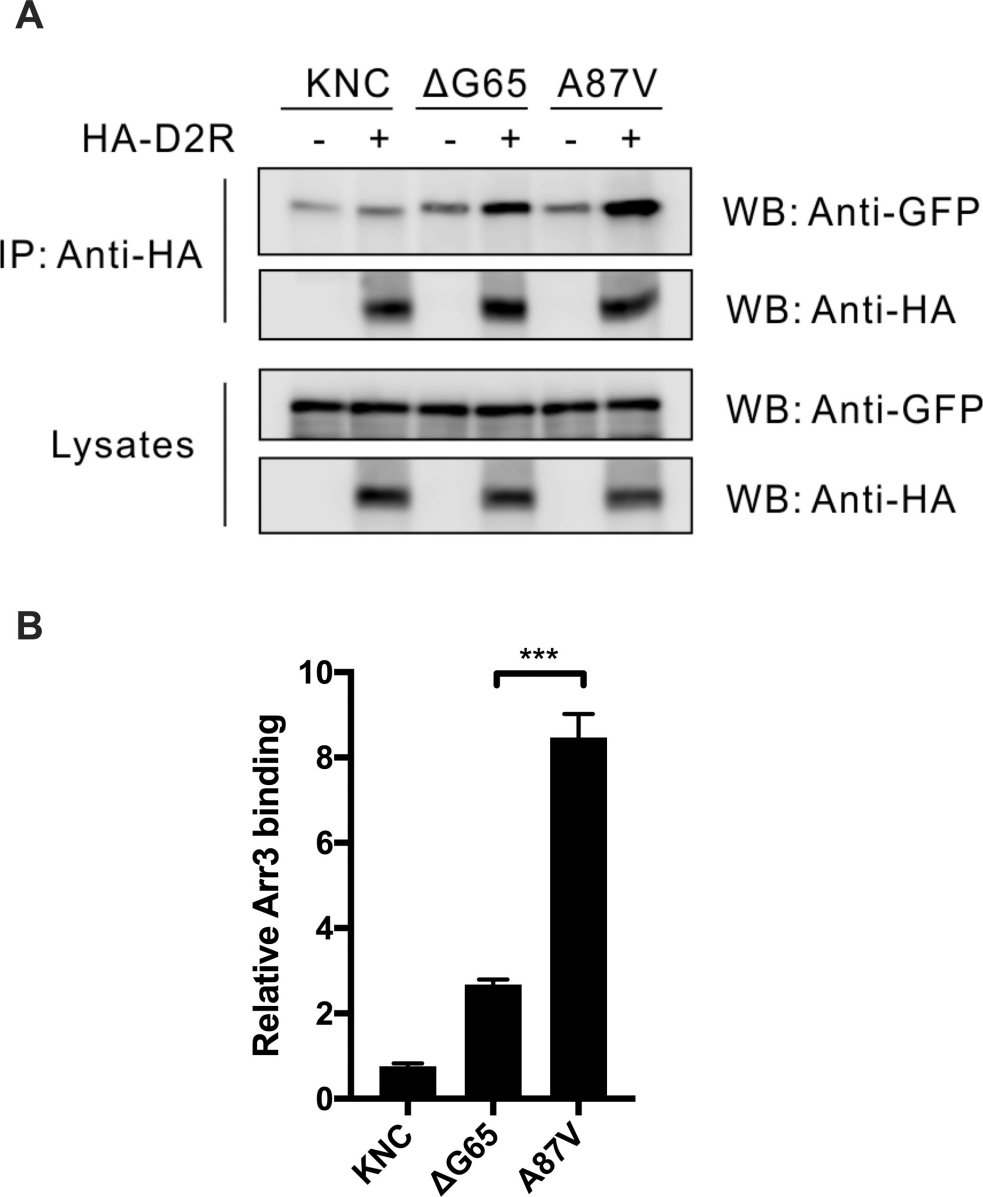
Deletion of G65 impedes arrestin-3 binding to D2R *in vitro*. **(A)**. Serum-starved HEK293 arrestin 2/3 KO cells were co-transfected with indicated Ve-Arr3 mutants with or without (no bait control) HA- D2R. Cells were stimulated with 10 μM quinpirole. Cleared cell lysates were immunprecipitated with HA antibody, and immunoblotted with anti-HA and anti-GFP antibodies. **(B)** Quantification of the specific receptor binding of different arrestin-3 mutants in three independent experiments. Non-specific binding observed in cells that do not express HA -D2R was subtracted in all cases.

As our in-cell BRET studies of the interaction of arrestin-3 mutants with GPCRs yielded surprising results, we sought to test these interactions by an independent method. We used co-immunoprecipitation (co-IP) of receptors with various forms of arrestin-3. HEK293 arrestin 2/3 KO cells transfected with Venus-tagged arrestin-3 mutants without HA-tagged D2R served as control for non-specific arrestin binding to the beads. Cells were co-transfected with indicated forms of Ve-Arr3 and HA-D2R. Following stimulation with the agonist, cell lysates were immunoprecipitated with anti-HA antibody and immunoblotted with anti-GFP and anti-HA antibodies (Fig 4). As shown in Fig 3A, upon stimulation KNC has almost no ability to bind the receptor, as compared to fully functional A87V base mutant. The deletion of G65 dramatically reduced arrestin-3 binding to D2R (Fig 3B). The same changes were observed by co-IP (Fig 4), confirming BRET results.

## Discussion

Mutations in numerous proteins underlie a variety of human disorders. Mutations in GPCRs fall into two categories: loss- and gain-of-function [11,12]. Despite the initial enthusiasm about editing errors out of the genome, careful studies show that CRISPR/Cas-9 gene editing often generates off-target changes, some of which could be expected, whereas others could not [45]. In more traditional gene therapy approach, the strategy in case of loss-of-function mutations is clear: the delivery of the coding sequence for the normal receptor should solve the problem. Loss-of-function mutations are usually recessive, as one normal allele is in most cases sufficient, so that only compound heterozygotes are affected. In contrast, gain-of-function mutations are dominant: normal product of the second allele cannot dampen the signaling by an overactive mutant receptor. One strategy was proposed to fight gain-of-function GPCR mutations: dampening their excessive signaling with enhanced arrestins that have higher propensity to bind these receptors. So far this compensational approach had shown promise in the visual system, where the only important receptor is rhodopsin, which is shut off by arrestin-1 [13,46]. While due to highly conserved activation mechanisms non-visual arresins can be enhanced by the same mutations as visual arrestin-1 [47–49], non-visual arrestins have broad receptor specificity [15,50,51]. Most cells express multiple GPCR subtypes. While the expression of enhanced non-visual arrestin would likely dampen excessive signaling by the mutant, at the same time it would reduce the signaling of perfectly normal other GPCRs in the same cell. Thus, the use of the same compensational approach requires non-visual arrestin variants with narrow receptor specificity, that would target only the “offending” receptor mutant, but not the other GPCRs.

Arrestins in mammals are represented by only four subtypes [7,8], so that the two non-visual arrestins control hundreds of different GPCRs [12]. However, arrestin-1 shows high specificity toward one receptor (rhodopsin), suggesting that narrow receptor specificity is possible [14,51]. Structural analysis of arrestins suggests similarity of the mechanism of receptor binding [29–31,33,52]. Arrestin-3 has been reported to bind virtually every GPCR tested [33,50], often demonstrating higher affinity for the receptors that both non-visual subtypes bind [50,53]. Thus, arrestin-3 appears to be a more promising starting point for the design of receptor-specific mutants than highly homologous arrestin-2 [54,55]. Previously, we have successfully changed the arrestin-3 specificity for particular GPCRs through manipulating relatively few residues on the receptor-binding surface [14,15,22,35]. Here we tested several mutations in the loops of arrestin-3 that are expected to engage the receptors using in-cell BRET assay.

The results clearly show that G65 in the finger loop is required for arrestin-3 binding to all tested receptors: M2R, D1R, D2R, β_2_AR. The deletion of G65 had the greatest effect on D2R binding (Fig 2). D2R, in contrast to D1R and β_2_AR, has a long third ICL [56,57]. However, it shares this structural feature with M2R [58], where the reduction of binding was similar to that of D1R and β_2_AR, so that the size of the third ICL per se (and likely consequent different “pose” of bound arrestin), does not explain greater suppression of the D2R binding. Other mutations (G65P, E157A, F245A and K313A) do not significantly change arrestin-3 binding to these GPCRs. Available crystal structures show that the finger loop of arrestin can be presented to the receptor in distinct conformations and at different angles (relative to the rest of the molecule) [41,43], suggesting that the flexibility of finger loop is important to allow the recognition of different receptors by non-visual arrestins. The mutations that perturb the flexibility of the finger loop were shown to interfere with receptor binding [41]. In particular, substitution of several residues with a rigid proline (D68P, D70P) has been shown to greatly decrease arrestin-3 binding to M2R and D2R [41]. Substitution of many residues by alanines generally decreases the binding of arrestin-1 to rhodopsin [21,59]. Unlike these mutations, ΔG65 and G65P differentially affect arrestin-3 binding without altering the charges in the finger loop. Glycine is the most sterically flexible residue among all amino acids due to its smallest side chain. The absence of this glycine apparently “tightens” the finger loop and/or allows an extension of the β-strand V, as Gly is known to break the secondary structure. Both mechanisms would explain uniformly negative effect of its deletion on GPCR binding (Fig 2).

The replacement of glycine with proline not only eliminates the flexibility of finger loop, but also bends the loop to a fixed angle due to the cyclic structure of the Pro residue. Our data show that G65P does not significantly affect the binding to the four receptors tested, suggesting that the interruption of the strand even by a sharp turn allows finger loop to accommodate these receptors. Considering that a glycine in position equivalent to G65 in arrestin-3 is conserved in virtually all arrestins [7,8], both high flexibility and proper length of the finger loop seem to be very important for arrestin function. Consequently, it is possible that G65P may impair arrestin-3 binding to other GPCRs, narrowing the broad receptor specificity of arrestin-3. This needs to be tested experimentally.

Arrestins appear to independently recognize two features in a GPCR: its active conformation and the presence of receptor-attached phosphates [60] (this mechanism was reviewed and illustrated in [5]). Recent findings suggest that, at least in case of GPCRs where all phosphorylation sites are localized on the receptor C-terminus, arrestin binds to just the phosphorylated elements tightly enough to yield stable receptor complexes with the “partially engaged” arrestins [61–63]. Our data indicate that the deletion of G65 in the finger loop, which engages non-phosphorylated parts of the receptor, the inter-helical cavity that opens upon GPCR activation [16,17,64], has a very strong negative effect on the arrestin-receptor interaction. As ΔG65 mutation is unlikely to directly affect arrestin-3 interactions with the receptor-attached phosphates directly, it appears that mutant finger loop weakens arrestin-3 binding even to the phosphorylated receptor elements indirectly, via an allosteric mechanism. Interestingly, D2 receptor, where the effect is the strongest, is a special case: in it GRK phosphorylation sites are localized exclusively on the ICL3, and phosphorylation does not seem to be required for the arrestin-3 binding [19]. Although GRK phosphorylation sites are also localized on the ICL3 in the M2 muscarinic receptor [58], in this case phosphorylation is necessary for the arrestin binding [58,65]. Thus, the magnitude of the effect of ΔG65 mutation on arrestin-3 interaction with tested GPCRs might reflect relative contribution of the arrestin binding to the receptor-attached phosphates: the greater the role of phosphate binding (which is very different in GPCR subtypes used here [20,26]), the smaller the effects of the mutation that does not affect phosphate-binding arrestin elements identified in several studies [41,66,67].

E157 and K313 are charged residues. In fact, K313 is the only positively charged amino acid in the back loop. The common strategy of substitution is to change to oppositely charged amino acid to check the role of this residue. We constructed E157K and K313E mutants with charge reversals and found that both showed very low expression level in cells, suggesting that these mutants have folding problems. In contrast, charge elimination mutations E157A and K313A appear to be well tolerated (Fig 1).

To summarize, our data indicate that the absence of a highly flexible glycine and/or the length of the finger loop, which likely affects its ability to “mold” itself to fit the receptor in the complex, plays a more important role in receptor binding than the actual sequence in other elements. This is consistent with the interaction of the finger loop with the inter-helical cavity in the core of active GPCRs, revealed by the structure of the arrestin-1 complex with rhodopsin [16,17], supporting the idea that this element plays the same critical role in the binding of non-visual arrestins to other GPCRs.

## Supporting information

S1 Fig Expression of Venus-arrestin and receptor-*R*luc8. The expression of Venus-arrestin and receptor-*R*Luc8 were evaluated by the fluorescence and the basal luminescence density, respectively. The expression level was normalized to the A87V base mutant, as follows: M2R (A), β_2_AR(**B**), D1R(**C**), D2R(**D**).
(PDF)

## References

[1] Bockaert J, Pin JP (1999) Molecular tinkering of G protein-coupled receptors: an evolutionary success. EMBO J 18: 1723–1729. 10.1093/emboj/18.7.1723 10202136 PMC1171258

[2] Lagerstrom MC, Schioth HB (2008) Structural diversity of G protein-coupled receptors and significance for drug discovery. Nat Rev Drug Discov 7: 339–357. 10.1038/nrd2518 18382464

[3] Fredriksson R, Lagerstrom MC, Lundin LG, Schioth HB (2003) The G-protein-coupled receptors in the human genome form five main families. Phylogenetic analysis, paralogon groups, and fingerprints. Mol Pharmacol 63: 1256–1272. 10.1124/mol.63.6.1256 12761335

[4] Gurevich EV, Tesmer JJ, Mushegian A, Gurevich VV (2012) G protein-coupled receptor kinases: more than just kinases and not only for GPCRs. Pharmacol Ther 133: 40–46. 10.1016/j.pharmthera.2011.08.001 21903131 PMC3241883

[5] Gurevich VV, Gurevich EV (2004) The molecular acrobatics of arrestin activation. Trends Pharmacol Sci 25: 105–111. 10.1016/j.tips.2003.12.008 15102497

[6] Carman CV, Benovic JL (1998) G-protein-coupled receptors: turn-ons and turn-offs. Curr Opin Neurobiol 8: 335–344. 9687355 10.1016/s0959-4388(98)80058-5

[7] Gurevich EV, Gurevich VV (2006) Arrestins are ubiquitous regulators of cellular signaling pathways. Genome Biol 7: 236 10.1186/gb-2006-7-9-236 17020596 PMC1794542

[8] Indrischek H, Prohaska SJ, Gurevich VV, Gurevich EV, Stadler PF (2017) Uncovering missing pieces: duplication and deletion history of arrestins in deuterostomes. BMC Evol Biol 17: 163 10.1186/s12862-017-1001-4 28683816 PMC5501109

[9] Hauser AS, Attwood MM, Rask-Andersen M, Schioth HB, Gloriam DE (2017) Trends in GPCR drug discovery: new agents, targets and indications. Nat Rev Drug Discov 16: 829–842. 10.1038/nrd.2017.178 29075003 PMC6882681

[10] Thompson MD, Hendy GN, Percy ME, Bichet DG, Cole DE (2014) G protein-coupled receptor mutations and human genetic disease. Methods Mol Biol 1175: 153–187. 10.1007/978-1-4939-0956-8_8 25150870

[11] Schoneberg T, Schulz A, Biebermann H, Hermsdorf T, Rompler H, et al (2004) Mutant G-protein-coupled receptors as a cause of human diseases. Pharmacol Ther 104: 173–206. 10.1016/j.pharmthera.2004.08.008 15556674

[12] Stoy H, Gurevich VV (2015) How genetic errors in GPCRs affect their function: Possible therapeutic strategies. Genes Dis 2: 108–132. 10.1016/j.gendis.2015.02.005 26229975 PMC4516391

[13] Song X, Vishnivetskiy SA, Gross OP, Emelianoff K, Mendez A, et al (2009) Enhanced Arrestin Facilitates Recovery and Protects Rod Photoreceptors Deficient in Rhodopsin Phosphorylation. Curr Biol 19: 700–705. 10.1016/j.cub.2009.02.065 19361994 PMC2768495

[14] Vishnivetskiy SA, Gimenez LE, Francis DJ, Hanson SM, Hubbell WL, et al (2011) Few residues within an extensive binding interface drive receptor interaction and determine the specificity of arrestin proteins. J Biol Chem 286: 24288–24299. 10.1074/jbc.M110.213835 21471193 PMC3129209

[15] Gimenez LE, Vishnivetskiy SA, Baameur F, Gurevich VV (2012) Manipulation of very few receptor discriminator residues greatly enhances receptor specificity of non-visual arrestins. J Biol Chem 287: 29495–29505. 10.1074/jbc.M112.366674 22787152 PMC3436164

[16] Kang Y, Zhou XE, Gao X, He Y, Liu W, et al (2015) Crystal structure of rhodopsin bound to arrestin by femtosecond X-ray laser. Nature 523: 561–567. 10.1038/nature14656 26200343 PMC4521999

[17] Zhou XE, He Y, de Waal PW, Gao X, Kang Y, et al (2017) Structural Identification of Phosphorylation Codes for Arrestin Recruitment by G protein-Coupled Receptors. Cell 170: 457–469. 10.1016/j.cell.2017.07.002 28753425 PMC5567868

[18] Donthamsetti P, Quejada JR, Javitch JA, Gurevich VV, Lambert NA (2015) Using Bioluminescence Resonance Energy Transfer (BRET) to Characterize Agonist-Induced Arrestin Recruitment to Modified and Unmodified G Protein-Coupled Receptors. Curr Protoc Pharmacol 70: 2 14 11–14. 26331887 10.1002/0471141755.ph0214s70 PMC4583203

[19] Namkung Y, Dipace C, Urizar E, Javitch JA, Sibley DR (2009) G protein-coupled receptor kinase-2 constitutively regulates D2 dopamine receptor expression and signaling independently of receptor phosphorylation. J Biol Chem 284: 34103–34115. 10.1074/jbc.M109.055707 19815545 PMC2797181

[20] Namkung Y, Dipace C, Javitch JA, Sibley DR (2009) G protein-coupled receptor kinase-mediated phosphorylation regulates post-endocytic trafficking of the D2 dopamine receptor. J Biol Chem 284: 15038–15051. 10.1074/jbc.M900388200 19332542 PMC2685686

[21] Pfleger KD, Eidne KA (2006) Illuminating insights into protein-protein interactions using bioluminescence resonance energy transfer (BRET). Nat Methods 3: 165–174. 10.1038/nmeth841 16489332

[22] Prokop S, Perry NA, Vishnivetskiy SA, Toth AD, Inoue A, et al (2017) Differential manipulation of arrestin-3 binding to basal and agonist-activated G protein-coupled receptors. Cell Signal 36: 98–107. 10.1016/j.cellsig.2017.04.021 28461104 PMC5797668

[23] Alvarez-Curto E, Inoue A, Jenkins L, Raihan SZ, Prihandoko R, et al (2016) Targeted Elimination of G Proteins and Arrestins Defines Their Specific Contributions to Both Intensity and Duration of G Protein-coupled Receptor Signaling. J Biol Chem 291: 27147–27159. 10.1074/jbc.M116.754887 27852822 PMC5207144

[24] Grundmann M, Merten N, Malfacini D, Inoue A, Preis P, et al (2018) Lack of beta-arrestin signaling in the absence of active G proteins. Nat Commun 9: 341 10.1038/s41467-017-02661-3 29362459 PMC5780443

[25] Breitman M, Kook S, Gimenez LE, Lizama BN, Palazzo MC, et al (2012) Silent scaffolds: inhibition OF c-Jun N-terminal kinase 3 activity in cell by dominant-negative arrestin-3 mutant. J Biol Chem 287: 19653–19664. 10.1074/jbc.M112.358192 22523077 PMC3366000

[26] Gimenez LE, Kook S, Vishnivetskiy SA, Ahmed MR, Gurevich EV, et al (2012) Role of receptor-attached phosphates in binding of visual and non-visual arrestins to G protein-coupled receptors. J Biol Chem 287: 9028–9040. 10.1074/jbc.M111.311803 22275358 PMC3308753

[27] Gimenez LE, Babilon S, Wanka L, Beck-Sickinger AG, Gurevich VV (2014) Mutations in arrestin-3 differentially affect binding to neuropeptide Y receptor subtypes. Cell Signal 26: 1523–1531. 10.1016/j.cellsig.2014.03.019 24686081 PMC4033671

[28] Granzin J, Wilden U, Choe HW, Labahn J, Krafft B, et al (1998) X-ray crystal structure of arrestin from bovine rod outer segments. Nature 391: 918–921. 10.1038/36147 9495348

[29] Hirsch JA, Schubert C, Gurevich VV, Sigler PB (1999) The 2.8 A crystal structure of visual arrestin: a model for arrestin's regulation. Cell 97: 257–269. 10219246 10.1016/s0092-8674(00)80735-7

[30] Han M, Gurevich VV, Vishnivetskiy SA, Sigler PB, Schubert C (2001) Crystal structure of beta-arrestin at 1.9 A: possible mechanism of receptor binding and membrane Translocation. Structure 9: 869–880. 11566136 10.1016/s0969-2126(01)00644-x

[31] Milano SK, Pace HC, Kim YM, Brenner C, Benovic JL (2002) Scaffolding functions of arrestin-2 revealed by crystal structure and mutagenesis. Biochemistry 41: 3321–3328. 11876640 10.1021/bi015905j

[32] Sutton RB, Vishnivetskiy SA, Robert J, Hanson SM, Raman D, et al (2005) Crystal Structure of Cone Arrestin at 2.3Å: Evolution of Receptor Specificity. J Mol Biol 354: 1069–1080. 10.1016/j.jmb.2005.10.023 16289201

[33] Zhan X, Gimenez LE, Gurevich VV, Spiller BW (2011) Crystal structure of arrestin-3 reveals the basis of the difference in receptor binding between two non-visual subtypes. J Mol Biol 406: 467–478. 10.1016/j.jmb.2010.12.034 21215759 PMC3034793

[34] Ohguro H, Palczewski K, Walsh KA, Johnson RS (1994) Topographic study of arrestin using differential chemical modifications and hydrogen/deuterium exchange. Protein Sci 3: 2428–2434. 10.1002/pro.5560031226 7756996 PMC2142757

[35] Vishnivetskiy SA, Hosey MM, Benovic JL, Gurevich VV (2004) Mapping the arrestin-receptor interface. Structural elements responsible for receptor specificity of arrestin proteins. J Biol Chem 279: 1262–1268. 10.1074/jbc.M308834200 14530255

[36] Hanson SM, Gurevich VV (2006) The differential engagement of arrestin surface charges by the various functional forms of the receptor. J Biol Chem 281: 3458–3462. 10.1074/jbc.M512148200 16339758 PMC2440687

[37] Hanson SM, Cleghorn WM, Francis DJ, Vishnivetskiy SA, Raman D, et al (2007) Arrestin mobilizes signaling proteins to the cytoskeleton and redirects their activity. J Mol Biol 368: 375–387. 10.1016/j.jmb.2007.02.053 17359998 PMC1904837

[38] Pulvermuller A, Schroder K, Fischer T, Hofmann KP (2000) Interactions of metarhodopsin II. Arrestin peptides compete with arrestin and transducin. J Biol Chem 275: 37679–37685. 10.1074/jbc.M006776200 10969086

[39] Zhuang T, Chen Q, Cho M-K, Vishnivetskiy SA, Iverson TI, et al (2013) Involvement of Distinct Arrestin-1 Elements in Binding to Different Functional Forms of Rhodopsin. Proc Nat Acad Sci USA 110: 942–947. 10.1073/pnas.1215176110 23277586 PMC3549108

[40] Hanson SM, Gurevich VV (2006) The differential engagement of arrestin surface charges by the various functional forms of the receptor. J Biol Chem 281: 3458–3462. 10.1074/jbc.M512148200 16339758 PMC2440687

[41] Chen Q, Perry NA, Vishnivetskiy SA, Berndt S, Gilbert NC, et al (2017) Structural basis of arrestin-3 activation and signaling. Nat Commun 8: 1427 10.1038/s41467-017-01218-8 29127291 PMC5681653

[42] Gurevich VV, Gurevich EV (2014) Extensive shape shifting underlies functional versatility of arrestins. Curr Opin Cell Biol 27: 1–9. 10.1016/j.ceb.2013.10.007 24680424 PMC3971385

[43] Kim M, Vishnivetskiy SA, Van Eps N, Alexander NS, Cleghorn WM, et al (2012) Conformation of receptor-bound visual arrestin. Proc Natl Acad Sci U S A 109: 18407–18412. 10.1073/pnas.1216304109 23091036 PMC3494953

[44] Peterhans C, Lally CC, Ostermaier MK, Sommer ME, Standfuss J (2016) Functional map of arrestin binding to phosphorylated opsin, with and without agonist. Sci Rep 6: 28686 10.1038/srep28686 27350090 PMC4923902

[45] Anderson KR, Haeussler M, Watanabe C, Janakiraman V, Lund J, et al (2018) CRISPR off-target analysis in genetically engineered rats and mice. Nat Methods 15: 512–514. 10.1038/s41592-018-0011-5 29786090 PMC6558654

[46] Samaranayake S, Song X, Vishnivetskiy SA, Chen J, Gurevich EV, et al (2018) Enhanced Mutant Compensates for Defects in Rhodopsin Phosphorylation in the Presence of Endogenous Arrestin-1. Front Mol Neurosci 11: 203 10.3389/fnmol.2018.00203 29973866 PMC6020793

[47] Kovoor A, Celver J, Abdryashitov RI, Chavkin C, Gurevich VV (1999) Targeted construction of phosphorylation-independent b-arrestin mutants with constitutive activity in cells. J Biol Chem 274: 6831–6834. 10066734 10.1074/jbc.274.11.6831

[48] Celver J, Vishnivetskiy SA, Chavkin C, Gurevich VV (2002) Conservation of the phosphate-sensitive elements in the arrestin family of proteins. J Biol Chem 277: 9043–9048. 10.1074/jbc.M107400200 11782458

[49] Pan L, Gurevich EV, Gurevich VV (2003) The nature of the arrestin x receptor complex determines the ultimate fate of the internalized receptor. J Biol Chem 278: 11623–11632. 10.1074/jbc.M209532200 12525498

[50] Barak LS, Ferguson SS, Zhang J, Caron MG (1997) A beta-arrestin/green fluorescent protein biosensor for detecting G protein-coupled receptor activation. J Biol Chem 272: 27497–27500. 9346876 10.1074/jbc.272.44.27497

[51] Gurevich VV, Dion SB, Onorato JJ, Ptasienski J, Kim CM, et al (1995) Arrestin interaction with G protein-coupled receptors. Direct binding studies of wild type and mutant arrestins with rhodopsin, b2-adrenergic, and m2 muscarinic cholinergic receptors. J Biol Chem 270: 720–731. 7822302 10.1074/jbc.270.2.720

[52] Milano SK, Kim YM, Stefano FP, Benovic JL, Brenner C (2006) Nonvisual arrestin oligomerization and cellular localization are regulated by inositol hexakisphosphate binding. J Biol Chem 281: 9812–9823. 10.1074/jbc.M512703200 16439357

[53] Kohout TA, Lin FS, Perry SJ, C, D.A., Lefkowitz RJ (2001) beta-Arrestin 1 and 2 differentially regulate heptahelical receptor signaling and trafficking. Proc Natl Acad Sci U S A 98: 1601–1606. 10.1073/pnas.041608198 11171997 PMC29303

[54] Attramadal H, Arriza JL, Aoki C, Dawson TM, Codina J, et al (1992) Beta-arrestin2, a novel member of the arrestin/beta-arrestin gene family. J Biol Chem 267: 17882–17890. 1517224

[55] Sterne-Marr R, Gurevich VV, Goldsmith P, Bodine RC, Sanders C, et al (1993) Polypeptide variants of beta-arrestin and arrestin3. J Biol Chem 268: 15640–15648. 8340388

[56] Giros B, Sokoloff P, Martres MP, Riou JF, Emorine LJ, et al (1989) Alternative splicing directs the expression of two D2 dopamine receptor isoforms. Nature 342: 923–926. 10.1038/342923a0 2531847

[57] Monsma FJ Jr, McVittie LD, Gerfen CR, Mahan LC, Sibley DR (1989) Multiple D2 dopamine receptors produced by alternative RNA splicing. Nature 342: 926–929. 10.1038/342926a0 2480527

[58] Lee KB, Ptasienski JA, Pals-Rylaarsdam R, Gurevich VV, Hosey MM (2000) Arrestin binding to the M2 muscarinic acetylcholine receptor is precluded by an inhibitory element in the third intracellular loop of the receptor. J Biol Chem 275: 9284–9289. 10734068 10.1074/jbc.275.13.9284

[59] Ostermaier MK, Peterhans C, Jaussi R, Deupi X, Standfuss J (2014) Functional map of arrestin-1 at single amino acid resolution. Proc Natl Acad Sci U S A 111: 1825–1830. 10.1073/pnas.1319402111 24449856 PMC3918777

[60] Gurevich VV, Benovic JL (1993) Visual arrestin interaction with rhodopsin: Sequential multisite binding ensures strict selectivity towards light-activated phosphorylated rhodopsin. J Biol Chem 268: 11628–11638. 8505295

[61] Thomsen ARB, Plouffe B, Cahill TJ III, Shukla AK, Tarrasch JT, et al (2016) GPCR-G Protein-β-Arrestin Super-Complex Mediates Sustained G Protein Signaling. Cell 166: 907–919. 10.1016/j.cell.2016.07.004 27499021 PMC5418658

[62] Cahill TJ 3rd, Thomsen AR, Tarrasch JT, Plouffe B, Nguyen AH, et al (2017) Distinct conformations of GPCR-β-arrestin complexes mediate desensitization, signaling, and endocytosis. Proc Natl Acad Sci U S A 114: 2562–2567. 10.1073/pnas.1701529114 28223524 PMC5347553

[63] Kumari P, Srivastava A, Banerjee R, Ghosh E, Gupta P, et al (2016) Functional competence of a partially engaged GPCR-β-arrestin complex. Nat Commun 7: 13416 10.1038/ncomms13416 27827372 PMC5105198

[64] Szczepek M, Beyriere F, Hofmann KP, Elgeti M, Kazmin R, et al (2014) Crystal structure of a common GPCR-binding interface for G protein and arrestin. Nat Commun 5: 4801 10.1038/ncomms5801 25205354 PMC4199108

[65] Pals-Rylaarsdam R, Gurevich VV, Lee KB, Ptasienski J, Benovic JL, et al (1997) Internalization of the m2 muscarinic acetylcholine receptor: arrestin-independent and -dependent pathways. J Biol Chem 272: 23682–23689. 9295310 10.1074/jbc.272.38.23682

[66] Vishnivetskiy SA, Schubert C, Climaco GC, Gurevich YV, Velez M-G, et al (2000) An additional phosphate-binding element in arrestin molecule: implications for the mechanism of arrestin activation. J Biol Chem 275: 41049–41057. 10.1074/jbc.M007159200 11024026

[67] Gurevich VV, Benovic JL (1995) Visual arrestin binding to rhodopsin: diverse functional roles of positively charged residues within the phosphorylation-recignition region of arrestin. J Biol Chem 270: 6010–6016. 7890732 10.1074/jbc.270.11.6010

