# Supplementary material for: Critical role of the finger loop in arrestin binding to the receptors
Source: PLoS One. 2019 Mar 15;14(3):e0213792. doi: 10.1371/journal.pone.0213792 (PMC6420155; doi:10.1371/journal.pone.0213792)
Supplement: S1 Fig — The expression of Venus-arrestin and receptor-RLuc8 were evaluated by the fluorescence and the basal luminescence density, respectively. The expression level was normalized to the A87V base mutant, as follows: M2R (A), β2AR(B), D1R(C), D2R(D). (PDF) [file pone.0213792.s001.pdf]

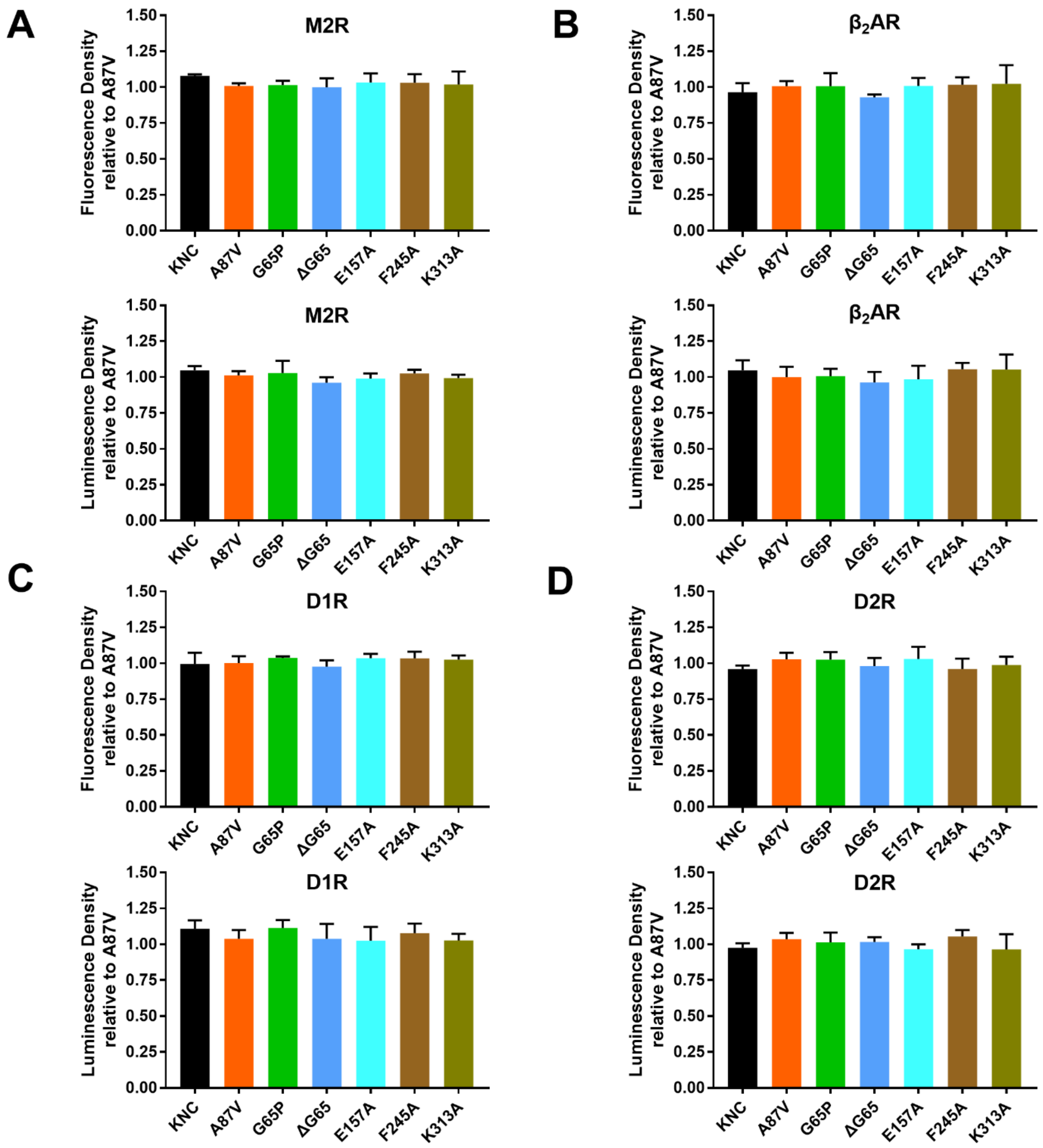

Suppl. Fig. 1. Expression of Venus-arrestin and receptor-*R*luc8. The expression of Venus-arrestin and receptor-*R*Luc8 were evaluated by the fluorescence and the basal luminescence density, respectively. The expression level was normalized to the A87V base mutant, as follows: M2R (A), β_2AR(**B**), D1R(**C**), D2R(**D**).
